# Retrospective study of patients with amelogenesis imperfecta treated with different bonded restoration techniques

**DOI:** 10.1002/cre2.243

**Published:** 2019-08-30

**Authors:** Hanne G. Ohrvik, Carl Hjortsjö

**Affiliations:** ^1^ Department of Prosthetic Dentistry and Oral Function, Faculty of Dentistry University of Oslo Oslo Norway

**Keywords:** amelogenesis imperfecta, ceramics, dental materials, esthetic, prosthodontics

## Abstract

**Objective:**

The aim of this retrospective study was to evaluate clinical success and satisfaction of patients with amelogenesis imperfecta treated with three different types of bonded restorations at a university clinic.

**Materials and Methods:**

One hundred fifty‐four restorations in 15 subjects with mean age of 17.3 years (*SD* 8.2) were evaluated after treatment with three different types of bonded restorations: all ceramic enamel‐dentin bonded restorations, prefabricated composite veneers, and direct composite resin restorations. A modified version of the Californian Dental Association system for quality evaluation of dental care and a questionnaire assessing patient satisfaction were used for classification. The restorations were evaluated with respect to patient satisfaction, esthetics, technical, and biological complications.

**Results:**

Mean observation period for the restorations was 42.5 months (*SD* 35.6). All restorations were in place at the time of the examination. Surface and color calibration showed a success of 95% for the ceramic enamel‐dentin bonded restorations, 44% for the direct composite resin restorations, and 0% for the prefabricated composite veneers. The same pattern was evident for anatomy and marginal integrity. The subjects reported a high degree of satisfaction with both the esthetics and function of their restorations.

**Conclusion:**

The results indicated that all ceramic restorations demonstrated the best results for patients with amelogenesis imperfecta.

## 1 INTRODUCTION

The term amelogenesis imperfecta (AI) refers to a heterogeneous group of genetic disorders characterized by defects in enamel formation of the teeth in the absence of any generalized or systemic diseases. This includes patients for whom a family history cannot be identified but where a mutation is present. It can affect all or some teeth in deciduous and/or permanent dentitions (Witkop, 1989). The prevalence varies from 1:700 in northern Sweden to 1:12,000–14,000 in United States (Bäckman & Holm, 1986; Hoppenreijs, Voorsmit, & Freihofer, 1998). This genetic disorder is known to be associated with malfunction of the enamel forming proteins ameloblastin, enamelin, tuftelin, and amelogenin (Hart et al., 2003).

Relative to dental enamel, four types of AI have been recognized clinically (Witkop, 1989). Type 1 is hypoplastic, with deficiency in the quantity of enamel. The mineralization of enamel appears to be normal, hard, and shiny; however, it is malformed. This is the most common type, found in 70% of the cases. Type 2 is hypomaturation; enamel appears mottled, opaque white to red‐brown coloration, is softer than normal, and tends to fracture from the underlying dentin. Type 3 is hypocalcified, characterized by a normal amount of poorly mineralized enamel. Type 4 is hypoplastic–hypomaturation enamel associated with taurodontism.

Management of each case of AI requires an interdisciplinary approach to obtain the best outcome. It requires personalized dental treatment plans, because several clinical problems are associated with the disease. Typical problems correlated with AI are dental hypersensitivity, caries, loss of vertical dimension due to rapid wear of the dentition, poor esthetic appearance, and increased accumulation of plaque deposits. Most patients require lifelong, extensive restorative care (Coffield et al., 2005). Negative psychological outcomes, due to compromised appearance and function, have been found to prejudice a person's attractiveness and reduce social interaction (Pousette Lundgren & Dahllöf, 2014).

Different treatment options have been proposed for treatment of AI‐affected teeth: from simple microabrasion in cases of hypomaturation AI to gold or stainless steel crowns, all ceramic crowns, porcelain laminate veneers, direct composite resin restorations (DCR), and onlays. Recently, the use of bonded restorations has gained popularity because of the many benefits associated with these materials: excellent esthetics, conservative tooth preparations, and improved wear resistance. These benefits make their use advantageous (Pousette Lundgren & Dahllöf, 2014; Sabatini & Guzmán‐Armstrong, 2009).

A recent systematic review of the literature stated that defining the most appropriate treatment option for patients with AI is difficult. There is currently no data available, at least in terms of evidence‐based dentistry, on what treatments are considered superior for rehabilitations of AI patients. Clinical performance of different materials and treatment modalities for AI patients is still based on case reports or case series (Dashash, Yeung, Jamous, & Blinkhorn, 2013). In another recent review, it was stated that there are few studies comparing and assessing different treatment options for AI patients (Sabandal & Schafer, 2016). There is however evidence that rehabilitation of AI patients with adhesively bonded crowns performed excellent with few complications (Klink, Groten, & Huettig, 2018; Pousette Lundgren, Morling Vestlund, & Dahllöf, 2018; Pousette Lundgren, Morling Vestlund, Trulsson, & Dahllöf, 2015).

The aim of this study was to evaluate the clinical success and patient satisfaction of AI patients treated with three different types of bonded restorations: all ceramic enamel‐dentin bonded restorations (CBR), prefabricated composite veneers (PCV), and DCR performed by postgraduate students at a university clinic. The purpose was to follow up the restoration material used on AI patients over time. The null hypothesis was that there is no difference in clinical performance between the different bonded restorations types.

## 2 MATERIAL AND METHODS

### 2.1 Study design

The study was approved by the Regional Committee for Medical and Health Research Ethics, South East Norway (REK sør‐øst 2017/1008).

The study design was a retrospective evaluation of patients treated with all ceramic enamel‐dentin bonded restorations, prefabricated composite veneers and direct composite resin restorations.

### 2.2 Subjects

All subjects were previously referred for dental treatment to the Department of Prosthetic Dentistry or the Department of Pedodontics Dentistry, University of Oslo (UiO), either in‐house or from general dental practitioners in the greater Oslo area. The subjects were treated by different postgraduate students of the departments between January 2007 and December 2017. The subjects had been referred to one of the clinics for the following treatment needs: hypersensitivity, loss of vertical dimension, change of tooth morphology, or for esthetic reasons.

An electronic search for patients was conducted in the computerized journal system (Salud Dental Suite, Dublin, Ireland) used at the Institute of Clinical Dentistry, UiO.

Search criteria were AI, ceramic crowns and ceramic veneers cemented with resin cements, PCV, and DCR, in the period between January 2007 and December 2017.

Inclusion criteria were AI patient treated with all CBR, PCV, and DCR treated at the Faculty of Dentistry, UiO.

Exclusion criteria were polycrystalline ceramics (e.g., zirkonia or aluminum oxide), conventional cemented restorations (i.e., nonenamel‐dentin bonded restorations and metal ceramic crowns).

One hundred fifty‐seven subjects were identified. Manual review of patient records excluded 130 subjects for not meeting inclusion criteria. Twenty‐seven subjects were contacted by telephone. Ten persons could not participate for various reasons: impossible to reach (*n* = 3), living abroad (*n* = 1), did not want to attend examination (*n* = 3), and received dental treatment in other clinics (*n* = 3). Seventeen subjects did volunteer to participate in the study and were given written information about the project. Eleven men and six women aged 8 to 38 years, with a mean age of 17.2 years were included. Informed consent was obtained prior to commencing the study. Two subjects were excluded after examination for not meeting inclusion criteria (treated with metal ceramic crowns; one male and one female). Fifteen subjects were included in the study with 154 restorations.

The participants were distributed as follows: four with hypoplastic type, three with hypomaturation type, five with hypocalcified type, and three were not given a classified type AI.

### 2.3 Registrations

This retrospective study was performed at the Department of Prosthetic Dentistry, UiO. The patient records of included subjects were reviewed, and the following data were registered: gender, age, location and type of restorations, material, and the function times of the restorations. The two first test subjects were examined with the two authors present, giving a state of consensus evaluation of the clinical parameters used. Only the first author examined the remaining subjects. Neither of the authors had performed any of the restorations themselves. The restorations were scored in accordance with the modified version of the Californian Dental Association (CDA) system for quality evaluation of dental care (Ryge, 1980), evaluating surface and color (CDA‐SC), anatomy (CDA‐anat), and marginal integrity (CDA‐marg) of each restoration. The CDA scores R = range of excellence, S = range of acceptability, T = replace or correct for prevention, and V = replace statim. The examination comprised a registration of a number of technical and biological data. The clinical examination also included bite‐wings and periapical radiographs.

Marginal fit (excellent, poor marginal integrity on X‐ray, probing defect without penetration, and visible evidence of irregularity with penetration on probing) and fractures and infractions of the restoration materials of all restorations were recorded.

Occlusal contacts between the restorations and the opposing teeth were recorded in the maximal intercuspal position, at lateral excursion and protrusive movements using an occlusion foil (Arti‐Fol 8 μ; Dr. Jean Bausch KG, Cologne, Germany).

Recordings at the abutment teeth included the amount of plaque, pocket depths, bleeding on probing (present or absent), and mobility. The amount of plaque was assessed according to a 4‐point scale by Silness and Löe (1964). Soft tissue around the restorations was evaluated in terms of color, shape, and appearance of the papilla.

Restorations with CDA scores excellent and acceptable were defined as success. They were free of all technical complications in the entire observation time. Restorations with CDA scores replace or correct for prevention and replace statim were defined as failure. Survival includes all restorations with CDA score excellent, acceptable, replace, or correct for prevention and replace statim. The restoration remained in situ with or without modifications over the entire observation period.

The subjects were asked to evaluate the appearance, function, comfort, and overall satisfaction with their restoration using a visual analogue scale (VAS; 0 = *not at all satisfied* to 100 = *extremely satisfied*).

### 2.4 Statistics

Descriptive statistics was used for presenting the data. The statistical analyses were performed using SPSS version 25 software (SPSS Inc., Chicago, IL, USA). Groups were compared using cross‐tabulation with chi‐square test. Kaplan–Meier test was used to estimate the mean survival time. Kaplan–Meier survival was calculated for each material group. The significance level was set at *P* < .05.

To avoid the dependency among restorations in the same patient, we used a random number generator (Excel 2016, Microsoft Corporation, Redmond, WA, USA) to select 15 restorations to represent one restoration in each subject (subject level). The results are based on all 154 restorations (restoration level) when relevant.

## 3 RESULTS

**Figure 1:**
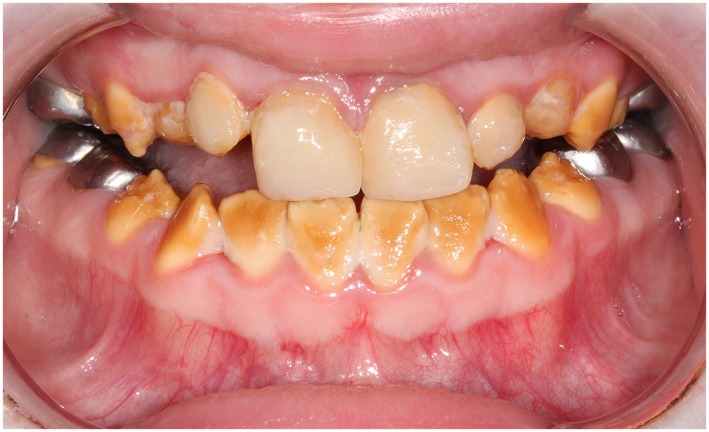
Eleven‐year‐old female with hypocalcification amelogenesis imperfecta. Direct composite resin restorations on teeth 12, 11, 21, and 22. Mean follow‐up time was 17 months

**Figure 2:**
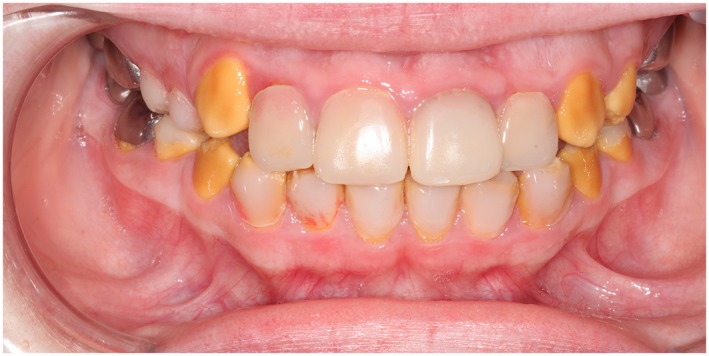
Fifteen‐year‐old female with hypocalcification amelogenesis imperfecta. Prefabricated composite veneers on teeth 12, 11, 21, and 22 and direct composite resin restorations on teeth 15, 14, 35, 33, 32, 31, 41, 42, 43, and 45. Mean follow‐up time for the prefabricated composite veneers was 35 months and 7 months for direct composite resin restorations

**Figure 3:**
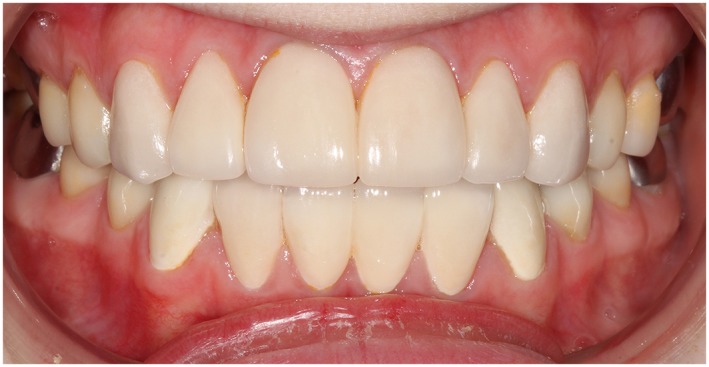
Eighteen‐year‐old female with hypoplastic amelogenesis imperfecta. Ceramic enamel‐dentin bonded restorations on teeth 15, 14, 13, 12, 11, 21, 22, 23, 24, 25, 35, 34, 33, 32, 31, 41, 42, 43, 44, and 45. Mean follow‐up time was 38 months

A total of 154 bonded restorations had been placed in 15 subjects: 44 DCR (Figure 1), eight PCV (Figure 2), and 102 CBR (Figure 3). The observation time of the restorations ranged from 1 month to 164 months, with a mean observation time of 42.5 months (*SD* = 35.6). The mean subject age of treatment and the mean subject age on which the restorations were scored is presented in Table 1.

**Table 1:** Distribution of restorations on types of amelogenesis imperfecta and mean age at the treatment and final examination, distribution of success and failure on restoration level, and distribution of restoration types on restoration and subject level

	Subjects (*n*)	Restoration level					Subject level		
		Success	Failure	DCR	PCV	CBR	DCR	PCV	CBR
**Type**									
Hypoplastic	4	30	21	16	0	35	2	0	2
Hypomaturation	3	13	9	4	2	16	1	1	1
Hypocalcified	5	19	27	20	6	20	3	1	1
Hypoplastic–hypomaturation	0	0	0	0	0	0	0	0	0
Not classified	3	29	6	4	0	31	1	0	2
Total	15	91	63	44	8	102	7	2	6
**Mean age (years)**									
Treamtent (*SD*)		18.0 (7.6)	16.0 (8.0)	12.2 (6.5)	13.0 (3.9)	19.7 (7.4)	10.1 (3.0)	11.5 (6.4)	20.0 (8.1)
Registration (*SD*)		22.3 (7.8)	18.6 (8.2)	14.1 (6.3)	15.5 (4.8)	24.1 (7.1)	12.1 (3.0)	13.5(7.8)	24.5 (7.7)

Abbreviations: CBR, ceramic enamel‐dentin bonded restorations; DCR, direct composite resin restorations; PCV, prefabricated composite veneers.

**Figure 4:**
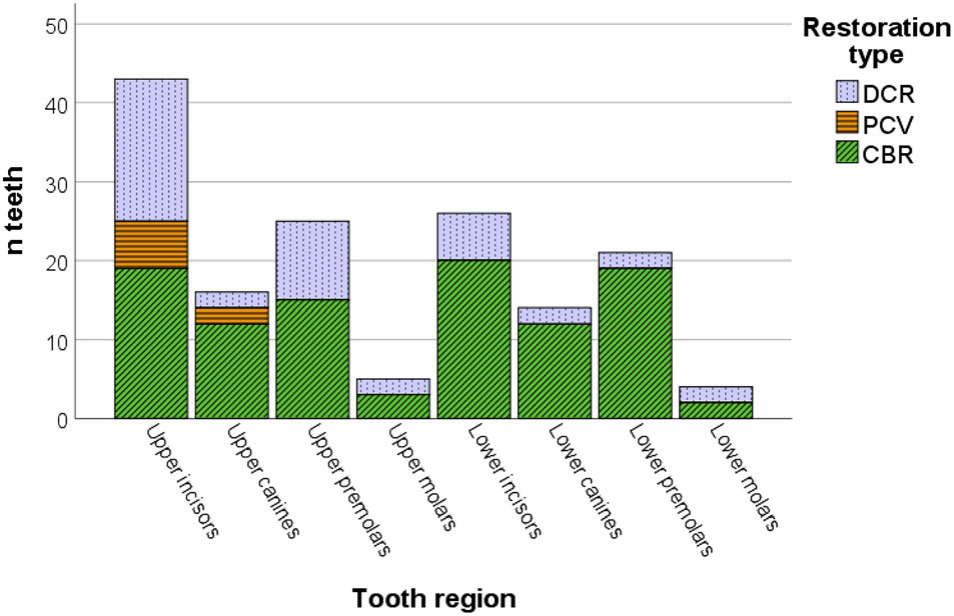
Tooth region distribution of DCR, PCV, and CBR restorations in amelogenesis imperfecta subjects. CBR, ceramic enamel‐dentin bonded restorations; DCR, direct composite resin restorations; PCV, prefabricated composite veneers

Three different dental restoration materials were used: (a) DCR, which included glass ionomer and composite (*n* = 44), (b) PCV (*n* = 8), and (c) CBR, which included Leucite reinforced press ceramic (Ivoclar Vivadent IPS Empress®, Schaan, Lichtenstein), lithium disilicate glass ceramic press (Ivoclar Vivadent IPS e.max®, Schaan, Lichtenstein), and feldspathic porcelain (*n* = 102). Distribution on restoration is presented in Figure 4 and Table 1.

All restorations were evaluated according to the CDA system (1977).

In CDA‐SC, 15 out of the 154 (9.7%) restorations were categorized as replace statim, of these six were PCV and nine DCR.

In CDA‐marg, 29 out of the 154 (19%) restorations were classified as replace or correct for prevention and 26 (17%) as replace statim. Eight PCV, 19 all CBR, and 28 DCR were all categorized as replace or correct for prevention or replace statim.

In CDA‐anat, 20 restorations were assessed as replace statim and 15 restorations as replace or correct for prevention. The data were replicated again, in that, PCV and DCR performed poorly. The data are presented on restoration and subject level in Table 2.

**Table 2:** CDA scores on restoration and subject level for the different material groups

CDA	Classification	Restoration level			Subject level		
		DCR	PCV	CBR	DCR	PCV	CBR
CDA‐SC^a^	Range of excellence	2 (5%)	0 (0%)	35 (34%)	0 (0%)	0 (0%)	1 (17%)
	Range of acceptability	17 (39%)	0 (0%)	62 (61%)	4 (57%)	0 (0%)	4 (67%)
	Replace or correct for prevention	16 (36%)	2 (25%)	5 (5%)	2 (28%)	0 (0%)	1 (17%)
	Replace statim	9 (20%)	6 (75%)	0 (0%)	1 (14%)	2 (100%)	0 (0%)
CDA‐anat^a^	Range of excellence	2 (5%)	0 (0%)	24 (24%)	0 (0%)	0 (0%)	1 (17%)
	Range of acceptability	19 (43%)	0 (0%)	74 (73%)	4 (57%)	0 (0%)	5 (83%)
	Replace or correct for prevention	10 (23%)	2 (25%)	3 (3%)	1 (14%)	0 (0%)	0 (0%)
	Replace statim	13 (30%)	6 (75%)	1 (1%)	2 (27%)	2 (100%)	0 (0%)
CDA‐marg^b^	Range of excellence	2 (5%)	0 (0%)	24 (24%)	0 (0%)	0 (0%)	1 (17%)
	Range of acceptability	14 (32%)	0 (0%)	59 (58%)	3 (43%)	0 (0%)	4 (67%)
	Replace or correct for prevention	10 (23%)	0 (0%)	19 (19%)	1 (14%)	0 (0%)	1 (17%)
	Replace statim	18 (41%)	8 (100%)	0 (0%)	3 (43%)	2 (100%)	0 (0%)

Abbreviations: CBR, ceramic enamel‐dentin bonded restorations; CDA, Californian Dental Association; DCR, direct composite resin restorations; PCV, prefabricated composite veneers.

Surface and color.

a Anatomic form.

b Marginal integrity.

Bleeding on probing was registered at 41 (27%) of the 154 restorations and mainly in teeth treated with PCV and DCR. No plaque was registered on 59 (38%) of the restorations, a thin layer on 72 (46%), a moderate amount on 19 (12%), and a large amount plaque on four (2%) restorations, respectively. The data are presented in Table 3.

**Table 3:** Biological and technical registrations on restoration and subject level

Registrations	Restoration level			Subject level		
	DCR	PCV	CBR	DCR	PCV	CBR
No plaque	13	3	43	2	0	2
Thin layer of plaque visible for the eye	19	3	50	3	1	4
Moderate amount of plaque	8	2	9	1	1	0
Large amount of plaque	4	0	0	1	0	0
Excellent marginal fit	14	0	58	3	0	2
Poor marginal integrity on x‐ray	4	0	19	1	0	2
Probing defect without penetration	14	0	25	1	0	2
Visible evidence of irregularity with penetration on probing	12	8	0	2	2	0
Bleeding present	16	5	20	2	2	0
Presence of fractures	0	0	2	0	0	0
Presence of infractions	0	0	2	0	0	0

Abbreviations: CBR, ceramic enamel‐dentin bonded restorations; DCR, direct composite resin restorations; PCV, prefabricated composite veneers.

The success rate at tooth level was 75.3% when looking at the CDA‐SC: 24% were excellent and 51.3% were acceptable. When looking at direct resin composite fillings, 4.5% of the total restorations were rated excellent, 38.6% as acceptable, and 36.4% were rated replace or correct for prevention. Among ceramic restorations, 34.3% were rated excellent, 60.8% as acceptable, and 4.9% were rated replace or correct for prevention. PCV achieved 25% of the replace or correct for prevention group, whereas we found zero observations in the excellent and acceptable groups.

In the CDA‐anat study, 77.3% of all observations at tooth level were characterized as success whereas 16.9% as excellent and 60.4% as acceptable. Among the direct composite resin fillings, 4.5% were rated as excellent, 43.2% as acceptable, and 22.7% as replace or correct for prevention.

Furthermore, the results from the ceramic study showed greater success as 23.5% were categorized excellent and 72.5% as acceptable. As in the CDA‐SC results, the prefabricated composite occurred as category replace or correct for prevention in 25% of the observations.

Looking at CDA‐marg, 64.3% were defined as success, 16.9% as excellent, and 47.4% as acceptable. Once again, the all CBR outperformed the other materials with 23.5% as excellent and 57.8% as acceptable. In the group of direct composite resin fillings, 4.5% were defined as excellent and 31.8% as acceptable. All of the PCV (100%) were rated as replace statim. Despite the poor rating, all restorations showed 100% survival. Cross‐tabulation and chi‐square test showed significant difference between the material groups, with respect to CDA‐SC, CDA‐anat, and CDA‐marg, and success on and restoration level in favor of CBR (*P* ˂ .001). On a subject level, there was no significant difference found (*P* = .083, *P* = .123, *P* = .227, and *P* = .252, respectively). Success was found in 14 out of the 44 (32%) DCR restorations, zero out of the eight (0%) PCV restorations, and 77 out of 102 (76%) CBR. On a subject level, the corresponding values were three out of seven (43%) in the DCR group, zero out of two (0%) in the PCV group, and four out of six (67%) in the CBR group. Cross‐tabulation and chi‐square test showed significant difference between the type of AI and success (*P* = .003; Table 1).

**Figure 5:**
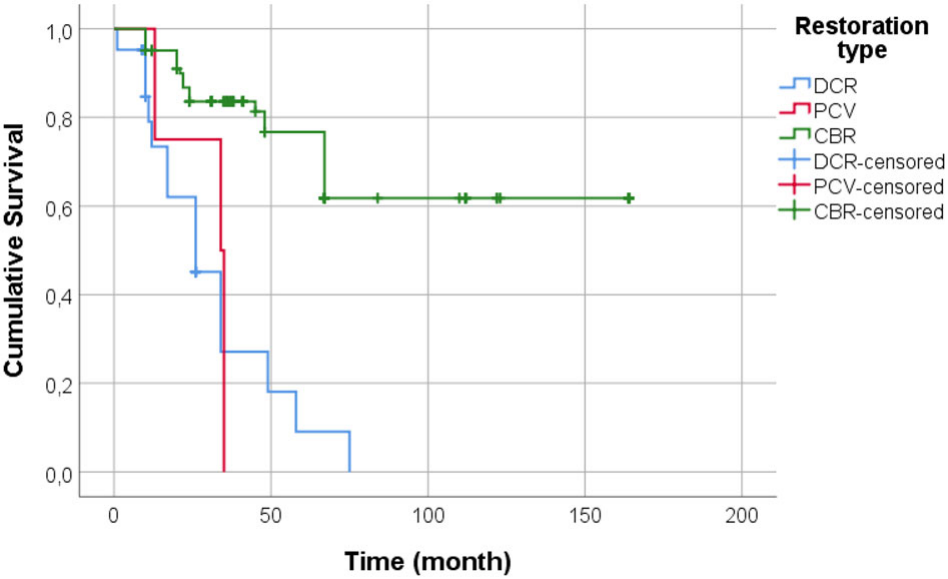
Kaplan–Meier plot showing longevity for DCR, PCV, and CBR restorations placed in subjects with amelogenesis imperfecta. CBR, ceramic enamel‐dentin bonded restorations; DCR, direct composite resin restorations; PCV, prefabricated composite veneers

Kaplan–Meier survival estimate showed a mean survival time of 31.3 months (SEM 3.8) for DCR, 29.3 months (SEM 3.5) for PCV, and 117.6 months (SEM 6.9) for CBR, respectively (Figure 5).

The mean value of the VAS assessment for the esthetics was 77.5, and for the restoration function 79.6. The patient's assessment of any possible problems with the restoration and their expectations of the dental treatment also demonstrated a high degree of satisfaction showing a mean value of the VAS assessment at 82.2.

## 4 DISCUSSION

This study aimed to evaluate the clinical success and patient satisfaction of AI patients treated with three different types of bonded restorations, DCR, PCV, and CBR, and to follow up these restorations over time. The null hypothesis was rejected; there was significant difference in clinical performance between the different bonded restorations types. The study showed that the use of PCV and direct resin fillings are poor alternatives compared with CBR. The Academy of Operative Dentistry, European section, suggests no direct resin restoration in severe AI cases. It was documented that in the presence of altered abnormal enamel, successful adhesive bonding may be difficult to achieve (Lynch, Opdam, Hickel, & Brunton, 2014). Case selection must be carefully considered when using direct‐bonded restorations. The greatest difficulties for bonding to enamel have been described in the AI hypocalcified type, which is characterized by a lower mineral content that seems to be deleterious to the bonding procedure (Faria‐e‐Silva et al., 2011; Saroglulu, Aras, & Öztas, 2006). This report showed a high failure rate in subjects with hypocalcified AI, 27 out the 46 restorations failed (59%) compared with the average failure rate of 63 out of the 154 restorations (40%). Poulsette Lundgren and Dahlöf concluded that the longevity of composite resin restorations was significantly lower in the AI group than in their control group. In the AI group, the 5‐year survival rate was 50%, compared with 80% in the control group. They also showed that the longevity of restorations was shorter and the quality was poorer in patients with hypomineralized/hypomaturated AI than in patients with hypoplastic AI (Pousette Lundgren & Dahllöf, 2014). Our study support this showing a low failure rate of 21 out the 51 hypoplastic AI subjects (41%).

We found that CBR scored highest on CDA‐SC, CDA‐anat, and CDA‐marg. In the CDA‐SC, 35 out of the 102 (34%) CBR were categorized as excellent. The restoration is of satisfactory quality and is expected to protect the tooth and the surrounding tissues, and of the 102 restorations, 62 (61 %) were classified as acceptable. In CDA‐anat, 24 CBR were assessed as excellent, and 74 were acceptable. There were obvious differences between the dental materials used looking at CDA‐marg: 24 CBR were excellent and 59 acceptable PCV had the weakest results, eight out of eight categorized as replace statim. Also, DCR showed a poor result, 18 out of 44 categorized as replace statim and 10 as replace or correct for prevention. They showed visible evidence of poor marginal integrity on X‐ray and probing defect with penetration. In these teeth, there were signs of discoloration and gingival inflammation.

The Kaplan–Meier should be interpreted with caution (DRC 31.3 months, PCV 29.3 months, and CBR 117.6 months, respectively) because even though failure, the restorations were defined as survived as they were in place at the time of examination, and an earlier careful examination would probably have revealed technical complications at an earlier stage. The technical complication associated with the PCV restorations has most likely been present from the start.

Shibata, Taguchi, Gondo, Stolf, and Baratieri (2016) compared ceramic veneers and direct composite in AI rehabilitation. They concluded that the selection criteria for the two different materials used in rehabilitation of AI patients depends on (a) disorder type and severity, (b) patient age, (c) esthetic demand, (d) treatment longevity, (e) presence or absence of parafunctional habits, (f????????) oral hygiene, and (g) financial cost.

Proper diagnosis and good treatment planning are fundamental to obtaining a satisfactory result for rehabilitation of patients with AI (Shibata et al., 2016).

There is general agreement, when the patient is in primary or mixed dentition, the main goal is to provide a treatment that can establish esthetics, chewing function, reduce dental hypersensitivity, and attrition until the patient approaches maturity, when a permanent treatment can be planned (Pires dos Santos, Cabral, Moliterno, & de Oliveira, 2008). Psychosocial factors should be considered; studies have reported lower self‐esteem, social avoidance, and negative psychological outcomes for people with AI (Pousette Lundgren & Dahllöf, 2014). Coffield et al. reported that more than 90% of subjects with AI felt tense or embarrassed about their teeth, and 60% said that life had been less satisfying because of problems with their teeth (Coffield et al., 2005). It is therefore encouraging to see our VAS study stating that 82.2% felt satisfied with their treatment, independent of the material chosen.

Worth noting in our study is the fact that the observations were skewed towards the CBR (102 restorations in six subjects) compared with DCR (44 restorations in seven subjects) and PCV (eight restorations in two subjects). The age when the treatment was performed was also skewed; CBR was performed on adolescents and adult individuals, in contrast to both DCR and PCV that were performed on children and adolescents. These two observations may add risk that the findings may appear more disfavorable for the latter two groups in particular for the PCV group. The data regarding PCV should be interpreted with caution.

DCR and PCV are mainly done on young individuals, and one cannot rule out that they were done as interim restorations; nevertheless, it is paramount that these restorations are free of both biological and technical complications and furthermore exhibit satisfying esthetics.

In recent years, the continuing development of all ceramic restorations have made it possible to create prosthetic restorations with minimally invasive techniques, high quality, and longevity (Pjetursson, Sailer, Makarov, Zwahlen, & Thoma, 2015). Today, all ceramic crowns made of leucite reinforced glass ceramic, lithium disilicate‐reinforced glass ceramics, or aluminum oxide‐based ceramics/zirconium dioxide can be recommended as treatment options in addition to the gold standard of metal ceramics for single crowns in anterior and posterior regions in adults (Saroglulu et al., 2006).

Lundgren et al. (2018) made a permanent therapy with ceramic crowns in young patients with AI, and they concluded that they could not find any differences between Procera and IPS e.max Press crowns. Survival after a mean period of 5.5 ± 0.8 years was 99.7% (Pousette Lundgren et al., 2018). Klink et al. (2018) followed up nine AI patients with adhesively bonded crowns up to 171 months (mean 76 and *SD* 44), and they calculated the annual failure rate to ˂1.5% and 10‐year success of 86%. They concluded that rehabilitation of AI patient with adhesively bonded crowns offers a long‐term survival and clinical success over time (Klink et al., 2018). Lundgren et al. (2018) concluded in a study on 27 AI patients aged between 11 and 22 years treated with porcelain crowns that prosthetic treatment should not be postponed (Pousette Lundgren et al., 2018). Our findings also support the views that patients with severe AI should be treated with permanent prosthetic restorations at an early age.

## 5 CONCLUSION

Within the limitations of this retrospective study, PCV should be avoided, and DCR may be used as interim restorative therapy when treating patients with AI. As a long‐term restorative therapy, CBR was considered first treatment modality of choice for both young and old AI patients.

## CONFLICT OF INTEREST

The authors do not have any financial interest in the companies whose materials are included in this article.

## CLINICAL SIGNIFICANCE

This retrospective clinical investigation compares the clinical and esthetic outcome of three different bonded restorations used in treating patients with amelogenesis imperfecta. Comparing prefabricated composite veneers, direct composite resin, and all ceramic bonded restorations, the ceramic enamel bonded restorations demonstrated the best clinical and esthetic outcome.
